# *PDX1* DNA Methylation Distinguishes Two Subtypes of Pancreatic Neuroendocrine Neoplasms with a Different Prognosis

**DOI:** 10.3390/cancers12061461

**Published:** 2020-06-04

**Authors:** Gitta Boons, Timon Vandamme, Joe Ibrahim, Geert Roeyen, Ann Driessen, Dieter Peeters, Ben Lawrence, Cristin Print, Marc Peeters, Guy Van Camp, Ken Op de Beeck

**Affiliations:** 1 Center for Oncological Research, University of Antwerp and Antwerp University Hospital, 2610 Antwerp, Belgium; gitta.boons@uantwerpen.be (G.B.); timon.vandamme@uantwerpen.be (T.V.); joe.ibrahim@uantwerpen.be (J.I.); marc.peeters@uza.be (M.P.); guy.vancamp@uantwerpen.be (G.V.C.); 2 Center of Medical Genetics, University of Antwerp and Antwerp University Hospital, 2650 Edegem, Belgium; 3 Section of Endocrinology, Department of Internal Medicine, Erasmus Medical Center, 3015GD Rotterdam, The Netherlands; 4 NETwerk, Antwerp University Hospital, 2650 Edegem, Belgium; 5 Department of Hepatobiliary, Endocrine and Transplantation Surgery, Antwerp University Hospital, 2650 Edegem, Belgium; geert.roeyen@uza.be; 6 Department of Pathology, Antwerp University Hospital and University of Antwerp, 2650 Edegem, Belgium; ann.driessen@uza.be; 7 Histopathology, Imaging and Quantification Unit, HistoGeneX, 2610 Antwerp, Belgium; dieter-peeters@telenet.be; 8 Department of Pathology, AZ Sint-Maarten, 2800 Mechelen, Belgium; 9 Discipline of Oncology, Faculty of Medicine and Health Sciences, University of Auckland, Auckland 1023, New Zealand; b.lawrence@auckland.ac.nz; 10 Maurice Wilkins Centre Hosted by the University of Auckland, Auckland 1023, New Zealand; c.print@auckland.ac.nz; 11 Department of Molecular Medicine and Pathology, School of Medical Sciences, Faculty of Medicine and Health Sciences, University of Auckland, Auckland 1023, New Zealand

**Keywords:** pancreatic neuroendocrine neoplasms, DNA methylation, PDX1, subtypes, cell-of-origin, prognosis

## Abstract

DNA methylation is a crucial epigenetic mechanism for gene expression regulation and cell differentiation. Furthermore, it was found to play a major role in multiple pathological processes, including cancer. In pancreatic neuroendocrine neoplasms (PNENs), epigenetic deregulation is also considered to be of significance, as the most frequently mutated genes have an important function in epigenetic regulation. However, the exact changes in DNA methylation between PNENs and the endocrine cells of the pancreas, their likely cell-of-origin, remain largely unknown. Recently, two subtypes of PNENs have been described which were linked to cell-of-origin and have a different prognosis. A difference in the expression of the transcription factor PDX1 was one of the key molecular differences. In this study, we performed an exploratory genome-wide DNA methylation analysis using Infinium Methylation EPIC arrays (Illumina) on 26 PNENs and pancreatic islets of five healthy donors. In addition, the methylation profile of the *PDX1* region was used to perform subtyping in a global cohort of 83 PNEN, 2 healthy alpha cell and 3 healthy beta cell samples. In our exploratory analysis, we identified 26,759 differentially methylated CpGs and 79 differentially methylated regions. The gene set enrichment analysis highlighted several interesting pathways targeted by altered DNA methylation, including MAPK, platelet-related and immune system-related pathways. Using the *PDX1* methylation in 83 PNEN, 2 healthy alpha cell and 3 healthy beta cell samples, two subtypes were identified, subtypes A and B, which were similar to alpha and beta cells, respectively. These subtypes had different clinicopathological characteristics, a different pattern of chromosomal alterations and a different prognosis, with subtype A having a significantly worse prognosis compared with subtype B (HR 0.22 [95% CI: 0.051–0.95], *p* = 0.043). Hence, this study demonstrates that several cancer-related pathways are differently methylated between PNENs and normal islet cells. In addition, we validated the use of the *PDX1* methylation status for the subtyping of PNENs and its prognostic importance.

## 1. Introduction

Pancreatic neuroendocrine neoplasms (PNENs) are rare neoplasms that can develop in the context of familial syndromes (10%). However, the vast majority of PNEN are sporadic occurrences (90%) [1,2]. The highest risk of developing PNENs is seen in the familial multiple endocrine neoplasia 1 (MEN1) syndrome, in which 60% of patients develop a PNEN due to germline mutations in the *MEN1* gene [3]. Other syndromes are linked to germline mutations in *VHL*, *CDKN1B*, *NF1*, *TSC1* and *TSC2* [4,5,6,7]. A growing body of evidence contributes to the understanding of molecular mechanisms in sporadic PNENs with the most frequently mutated genes being *MEN1* (44%), *DAXX* (25%), *ATRX* (18%) and PI3K-AKT-mTOR pathway genes (15%) [8]. Further unravelling of the pathways involved in PNENs has shown that the chromatin remodeling pathway and telomere maintenance pathway are frequently deregulated due to inactivation of *MEN1*, *ATRX*, *DAXX*, *SETD2* and *MLL3* [9]. As these pathways are important for epigenetic regulation, their frequent inactivation suggests an important role for epigenetic deregulation in PNEN tumorigenesis. So far, however, our understanding of the epigenetic landscape of PNENs remains incomplete, with DNA methylation being the most studied epigenetic feature [10,11,12].

PNENs can be classified as functional (25%) or non-functional PNENs (75%), based on, respectively, the presence or absence of symptoms associated with the overproduction of specific hormones [13]. The production of these specific hormones could be suggestive for cell-of-origin. Insulinomas (functional PNENs overproducing insulin), for example, are expected to originate from beta cells [14]. Non-functional PNENs most likely originate from different cell types, which might contribute to the observed heterogeneity in clinical characteristics and prognosis in this patient population. Our knowledge regarding cell-of-origin of, especially non-functional, PNENs remains limited [14]. However, two recent publications made important contributions [15,16]. As previously described, PNENs are frequently mutated in *DAXX*, *ATRX* and *MEN1* and mutations in these genes have been linked to prognosis [11]. Chan et al. compared the gene expression and DNA methylation profiles of tumors that are *ATRX/DAXX/MEN1* (A-D-M) mutated or A-D-M wild type (WT) [15]. They found that mutated and WT tumors clustered separately based on expression and DNA methylation profiles, with mutant tumors having an expression profile similar to alpha cells. Interestingly, in these mutant tumors, they described a high ARX and low PDX1 expression compared with WT tumors and a hypermethylation of four CpGs in the *PDX1* promotor of the mutant tumors [15]. Neiman et al. also found low DNA methylation levels in the *PDX1* promotor in beta cells and high DNA methylation levels in the *PDX1* promotor in alpha cells [17]. Furthermore, Cejas et al. performed subtyping of non-functional PNENs into two major subtypes, subtype A and subtype B, based on expression patterns and histone modifications in enhancer regions, which resemble alpha and beta cells, respectively [16]. Based on the H3K27 acetylation pattern, they also identified super-enhancer regions, which contain genes that play an important role in defining cell identity [16,18]. The *ARX* and *PDX1* regions met super-enhancer criteria in subtype A and B PNENs, respectively. Interestingly, subtyping was also possible using immunohistochemistry for ARX and PDX1 and these subtypes were associated with a significant difference in relapse-free survival [16].

Knowledge regarding genome-wide DNA methylation changes that occur during PNEN tumorigenesis is still limited. To further explore this, we have performed DNA methylation profiling of 26 PNENs and compared this to the methylation profile of five pancreatic islet cells using Infinium EPIC DNA methylation arrays (Illumina), which interrogate an extensive amount of CpGs. In addition, *PDX1* DNA methylation has not been used previously to perform PNEN subtyping, although PDX1 expression has been highlighted as one of the most important differences between recently described subtypes. To evaluate the potential of *PDX1* DNA methylation to perform subtyping and as a prognostic marker, we have combined our EPIC array data with additional Illumina 450K array data, resulting in an extensive cohort of 83 PNENs.

## 2. Results

### 2.1. Patient Characteristics

For this study, 26 PNEN patients were included in Belgium, 15 from the biobank at the Antwerp University Hospital (biobank@UZA, Antwerp, Belgium; ID: BE71030031000) [19], 7 from the "biothèque" of the University of Liège (BUL)—University Hospital (CHU) Liège (Belgium) and 4 from the tumor bank of the University Hospital Brussels (UZ Brussel, Belgium) [20]. The clinical and pathological characteristics of this patient cohort are summarized in Table S1. Fifty percent of the included patients were female, the mean age at diagnosis was 58 ± 17 years, 27% had a functional tumor (five insulinomas and two gastrinomas), two patients had metastatic disease at diagnosis and 12, 13 and 1 patient(s) were WHO grade 1, 2 and 3, respectively. Pancreatic islet samples were derived from five healthy donors, three males and two females and their mean age was 59 ± 4 years, which is in the same range as the PNEN study cohort. Pancreatic islets were used as controls, as PNENs develop from the endocrine cells of the pancreas. Fresh frozen tumor tissue from the 26 Belgian PNEN patients and fresh frozen pancreatic islets from the five healthy donors were analyzed using Infinium MethylationEPIC arrays (Illumina) (EPIC study cohort).

**Figure 1:**
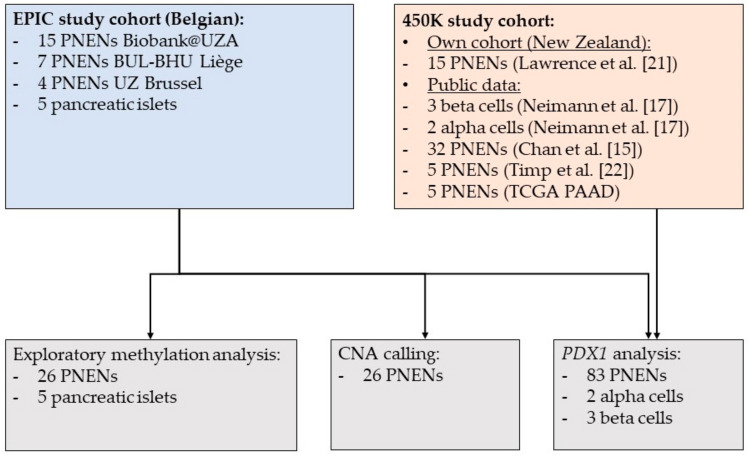
Overview of the study cohort and the analyses that were performed on (subsets of) the cohort. (CNA, copy number alteration; PNEN, pancreatic neuroendocrine neoplasm).

Infinium HumanMethylation450K BeadChip (Illumina) DNA methylation data of 15 additional fresh frozen samples of well-differentiated PNENs were generated in New Zealand [21]. Furthermore, Infinium HumanMethylation450K BeadChip (Illumina) DNA methylation data have been obtained via public databases from 32 PNENs analyzed by Chan et al. [15], 5 PNENs analyzed by Timp et al. [22] and 5 PNENs analyzed within the PAAD project of The Cancer Genome Atlas (TCGA) Research Network (https://www.cancer.gov/tcga). This resulted in 450K DNA methylation data of 57 PNENs (450K study cohort), of which, clinicopathological data were available for 52 (Table S1). Together with the EPIC study cohort, 83 patients were included, of which, 49% were female, the mean age at diagnosis was 56 ± 13 years, 82% had a nonfunctional tumor, 23 patients had described metastatic disease at diagnosis and 39, 30 and 3 patients were of WHO grade 1, 2 and 3, respectively. Figure 1 provides an overview of the included patients and the analyses that were performed on the different cohorts.

### 2.2. Exploratory DNA Methylation Analysis of EPIC Study Cohort

**Figure 2:**
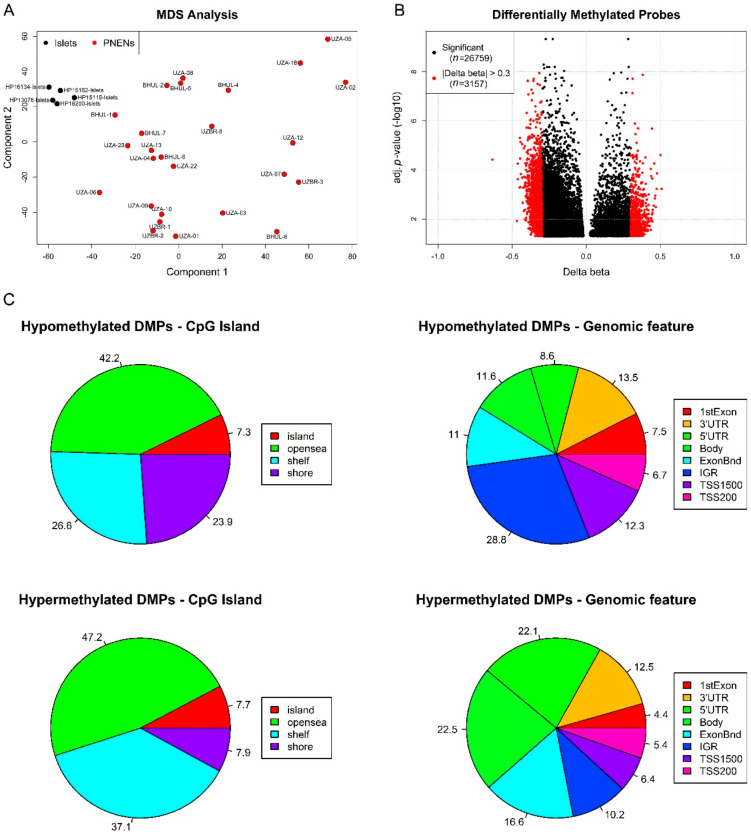
Exploratory analysis of EPIC DNA methylation data of 26 PNENs and 5 pancreatic islets. (**A**) Multidimensional scaling (MDS) analysis shows how PNENs (red) cluster separately from the pancreatic islets (black). (**B**) Volcano plot of all differentially methylated probes (DMPs) (black), the DMPs with a delta beta (difference in methylation between normal and tumor) greater than 0.3 are highlighted in red. (**C**) The distribution of hypo- and hypermethylated DMPs according to CpG island feature (island, open sea, shelf, shore) on the left and according to genomic feature (1^st^ exon, 3′ untranslated region (UTR), 5′UTR, body, exon boundary (ExonBnd), intergenic region (IGR), transcription start site (TSS) 1500 (200–1500 bases upstream of TSS) and TSS200 (0–200 bases upstream of TSS) on the right.

As a first step, we performed an exploratory DNA methylation analysis in the EPIC study cohort. For the main analysis, we have used the Chip Analysis Methylation Pipeline (ChAMP) package, using default settings unless indicated otherwise, which provides a pipeline that integrates available DNA methylation array analysis methods [23]. After read-in of the raw data, beta values representing the DNA methylation level were calculated. Beta values range from 0 (unmethylated) to 1 (completely methylated). As DNA methylation arrays use two probe technologies with a different dynamic range, normalization was performed and these normalized beta values were used for further analysis. Singular value decomposition (SVD) analysis was used to look at batch effects, but it identified no significant technical sources of variation in the data, so no correction had to be applied. The multidimensional scaling (MDS) analysis showed a dense clustering of the normal pancreatic islets, while the tumor samples are more spatially distributed (Figure 2A). ChAMP was also used to identify differential methylation between PNENs and pancreatic islets at individual CpGs (differentially methylated probes (DMPs)) or in regions (differentially methylated regions (DMRs)), with an adjusted *p*-value ≤ 0.05 as the cut-off of significance. The differential methylation analysis between PNENs and pancreatic islets identified 26,759 DMPs (Top 10 in Table S2) and 79 DMRs. Of the 26,759 DMPs, 14,480 could be mapped to 7213 genes, of which, 1487 genes contained 3 or more DMPs. An amount of 3157 DMPs had a difference in methylation (delta beta) larger than 0.3 (Figure 2B). Eighty-five percent of the DMPs are hypomethylated and the distribution of the hypermethylated and hypomethylated DMPs based on location in the CpG island and genomic feature is shown in Figure 2C. To allow both a comparison of hypo- versus hypermethylated DMPs and a comparison between the different categories, we have normalized the DMP counts by dividing them through the total number of analyzed CpGs in that category. The highest fraction of DMPs is situated in open sea regions (>4 kb from a CpG island), with comparable fractions for hypomethylated (42.2%) and hypermethylated (47.2%) DMPs. A higher fraction of hypomethylated DMPs (23.9%) is situated in CpG shore regions (0–2kb from CpG island) compared with hypermethylated DMPs (7.9%), while the fraction of hypermethylated DMPs (37.1%) in shelf regions (2–4 kb from CpG island) is higher than the fraction of hypomethylated DMPs (26.6%). Regarding the distribution of DMPs over genomic features, a few differences can be observed between hypo- and hypermethylated DMPs and between features. The highest portion of hypomethylated DMPs can be found in intergenic regions (28.8%), while the portion of all other features is less than 15%. Considering transcription start site (TSS) 1500 (200–1500 bases upstream of TSS), TSS200 (0–200 bases upstream of TSS) and 5′UTR as the promotor region, the fraction of hypermethylated DMPs (33.9%) is slightly higher than the fraction of hypomethylated DMPs (27.6%) in this promotor region. The highest portion of hypermethylated DMPs is present in the gene body (22.5%).

Gene set enrichment analysis, based on differential methylation analysis of individual CpGs, was performed using methylGSA [24]. This tool allows an assessment of an enrichment in differential methylation in certain gene sets, thereby adjusting for the number of CpGs in each gene to evade bias by the amount of CpGs analyzed per gene. Gene sets from Reactome, Gene Ontology (GO) and Kyoto Encyclopedia of Genes and Genomes (KEGG) pathways were interrogated. A significant enrichment was identified in 49 Reactome pathways, 549 GO categories and 7 KEGG pathways (Table S3). Among the most enriched Reactome pathways are MAPK-related pathways (e.g., “RAF/MAP kinase cascade”, “MAPK1/MAPK3 signaling” and “MAPK family signaling cascades”), death receptor signaling, platelet-related pathways and multiple immune-related pathways. Among the most enriched GO categories are chromatin/chromatid/centromere-related categories, “telomere maintenance”, “DNA damage checkpoint”, and several immune system/cytokine-related categories. Interestingly, within the seven significant KEGG pathways, “cytokine–cytokine receptor interaction” can be found in accordance with enrichment in immune system/cytokine-related gene sets in GO and Reactome. Furthermore, GO, KEGG and Reactome all indicated an enrichment of genes associated with olfactory receptor signaling.

As described, 79 DMRs were identified. The annotation of these regions and enrichment analysis were performed using the GREAT tool [25]. An enrichment was identified for the GO biological process “Regulation of immune system process” (false discovery rate (FDR) value = 0.046).

The enrichment in immune system components is not expected to result from the presence of immune cells as the tumor cell percentage is high in all tumor samples, although the exact impact of possibly present immune cells is difficult to quantify.

Next, we investigated whether epigenetic alterations in the immune system pathways resulted in immune hot or cold tumor sites. Therefore, we evaluated fresh frozen hematoxylin-eosin (HE)-stained slides of 18 evaluable PNENs for the presence of stromal tumor-infiltrating lymphocytes (TILs). The percentage of stromal TILs was low (≤ 5%) for most tumors, two tumors showed a slightly increased percentage of TILs (5%–20%) and only one sample (UZA-22) showed a higher percentage of stromal TILs of approximately 30% with clearly visible lymphoid aggregates (Table S1). Notably, the observed lymphoid aggregates can also originate from pre-existing lymphoid tissue as this sample originated from a lymph node metastasis, therefore it does not necessarily prove an immune activation by the tumor. These data suggest that altered methylation might result in the silencing of immune pathways and potentially enable the tumor to escape detection by the immune system.

### 2.3. PDX1 Methylation Status Distinguishes A and B Subtype

**Figure 3:**
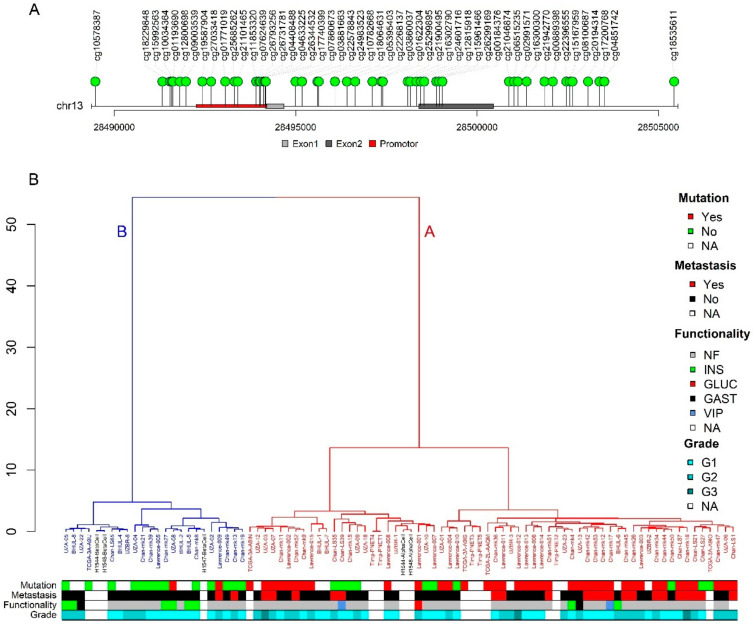
DNA methylation of the *PDX1* gene region can be used to perform subtyping. (**A**) Layout of the *PDX1* gene region with the CpG probes (green lollipops) that have been used for the clustering. (**B**) Unsupervised hierarchical clustering of the available PNEN, alpha cell (black) and beta cell (black) samples. Two groups can be distinguished, group A (in red), which contains the alpha cells and subtype A PNENs, and group B (in blue), which contains the beta cells and subtype B PNENs. Additional clinicopathological characteristics, e.g., mutation status of *ATRX/DAXX/MEN1*, presence of distance metastasis at diagnosis, functionality and WHO Grade (G1-3), are shown for the samples. (NA, not applicable or information not available; NF, non-functional; INS, insulinoma; GLUC, glucagonoma; GAST, gastrinoma; VIP, VIPoma).

As previously described, PDX1 is an important transcription factor in determining differentiation into pancreatic alpha or beta cells and might be useful to perform subtyping in PNENs. PDX1 is expressed in beta cells, while it is not expressed in alpha cells and *PDX1* has been reported to be hypermethylated in alpha cell-like PNENs [15]. *PDX1* has low methylation levels in beta cells and high methylation levels in alpha cells [17]. To study the potential of *PDX1* methylation to perform PNEN subtyping, we have combined all the collected PNEN methylation data and methylation data of alpha and beta cells. First, all the CpGs situated in the region of the *PDX1* gene, as described by Cejas et al. (chr13:28480000–28510000), and that are common between 450K and EPIC arrays, were selected (Figure 3A). Methylation values of the 83 PNENs, 2 alpha and 3 beta cells in this *PDX1* region were used to perform unsupervised hierarchical clustering (Figure 3B). The dendrogram shows two groups. In Figure 3B, we also indicated which tumors have mutations in *ATRX/DAXX/MEN1*, if this information was available, and other clinicopathological information. The biggest group contains 62 PNENs and almost all A-D-M mutated tumors. Interestingly, also the two alpha cell samples cluster within this group. This group of PNENs therefore corresponds to subtype A [15,16]. The other group consists of 21 PNENs which are almost exclusively A-D-M WT tumors and this group also contains beta cell samples, indicating that these are PNENs of subtype B.

### 2.4. Subtype Associates with Functionality and Metastatic Potential

Associations between the subtype and clinicopathological characteristics were tested and the results are summarized in Table 1. A significant association was found between subtype and mutation status, with a higher proportion of mutated tumors with subtype A, which is in agreement with the literature [15]. No significant association was found between WHO grade and subtype. However, all three G3 tumors were subtype A. Subtype and functionality of the tumor were significantly associated. A higher proportion of insulinomas was subtype B (*n* = 6) compared with subtype A (*n* = 2), the only glucagonoma was subtype A, gastrinomas were divided over the two subtypes, one with subtype A and one with subtype B and the two VIPomas were subtype A. Subtype was significantly associated with the presence of distant metastasis at diagnosis and showed a trend towards significant association with lymphovascular invasion (LVI) at diagnosis, with a higher proportion of cases with LVI and distant metastasis for subtype A compared with B. Recurrences after surgery were only observed in patients with subtype A PNENs, which resulted in a significant difference in recurrence between the two subtypes.

### 2.5. Subtypes Have a Different Prognosis

**Figure 4:**
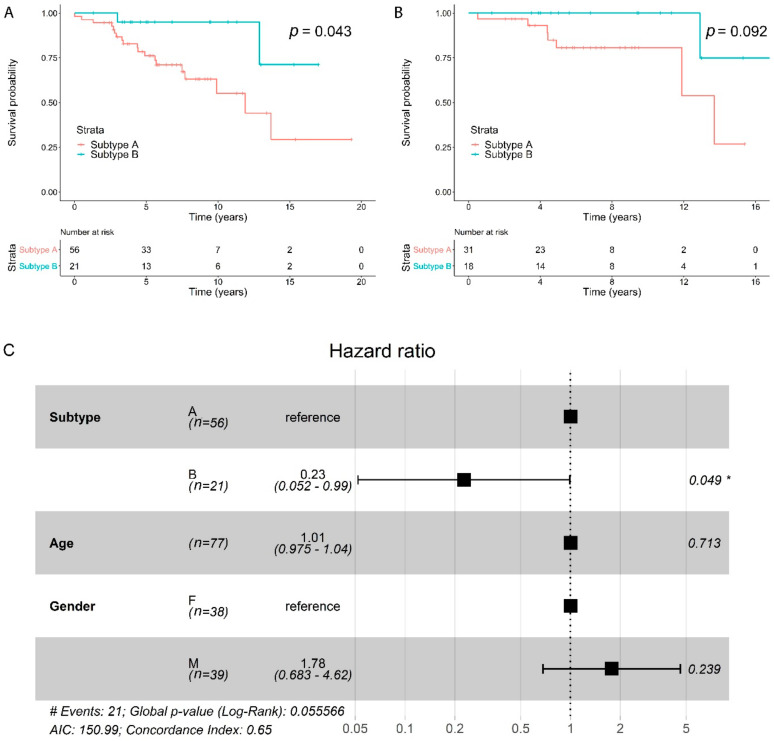
Survival analysis for the two subtypes, A and B. (**A**) Kaplan–Meier overall survival curve for all patients for which follow-up data were available with associated *p*-value from Cox proportional hazards analysis. (**B**) Kaplan–Meier overall survival curve for patients without distant metastasis at diagnosis with associated *p*-value from Cox proportional hazards analysis. (**C**) Cox proportional hazards model for subtype, age and gender (F, female; M, male). Hazards ratios with 95% confidence intervals and *p*-values are shown.

Overall survival analysis was performed using Cox proportional hazards (CoxPH) regression modeling, which investigates an association between survival time and predictor variables. Univariate CoxPH modeling for subtype showed that patients with subtype B had a significantly lower risk of death compared with subtype A with a hazards ratio (HR) of 0.22 for subtype B (95% confidence interval (CI): 0.051–0.95, *p* = 0.043). Figure 4A shows the associated Kaplan–Meier survival curve. Median overall survival for subtype A was 11.9 years (95% CI: 7.7 years—not reached) and was not reached for subtype B (95% CI: 12.9 years—not reached). The CoxPH analysis was repeated in a subpopulation of patients where there was no distant metastasis present at diagnosis, resulting in a HR of 0.16 for subtype B (95% CI: 0.019–1.4, *p* = 0.092) (Figure 4B). 

In a multivariate CoxPH model, which included both age and gender, a significant effect of subtype on overall survival was also observed with a HR of 0.23 for subtype B (95% CI: 0.052–0.99, *p* = 0.049) (Figure 4C). In a multivariate CoxPH model including distant metastasis and WHO grade besides subtype, age and gender, only distant metastasis (HR 3.58 [95% CI: 1.26–10.2], *p* = 0.017) and WHO grade 3 (HR 20.33 [95% CI: 3.90–106.1], *p* < 0.001) had a significant effect on survival.

### 2.6. Copy Number Alterations are Different between the Two Subtypes

**Figure 5:**
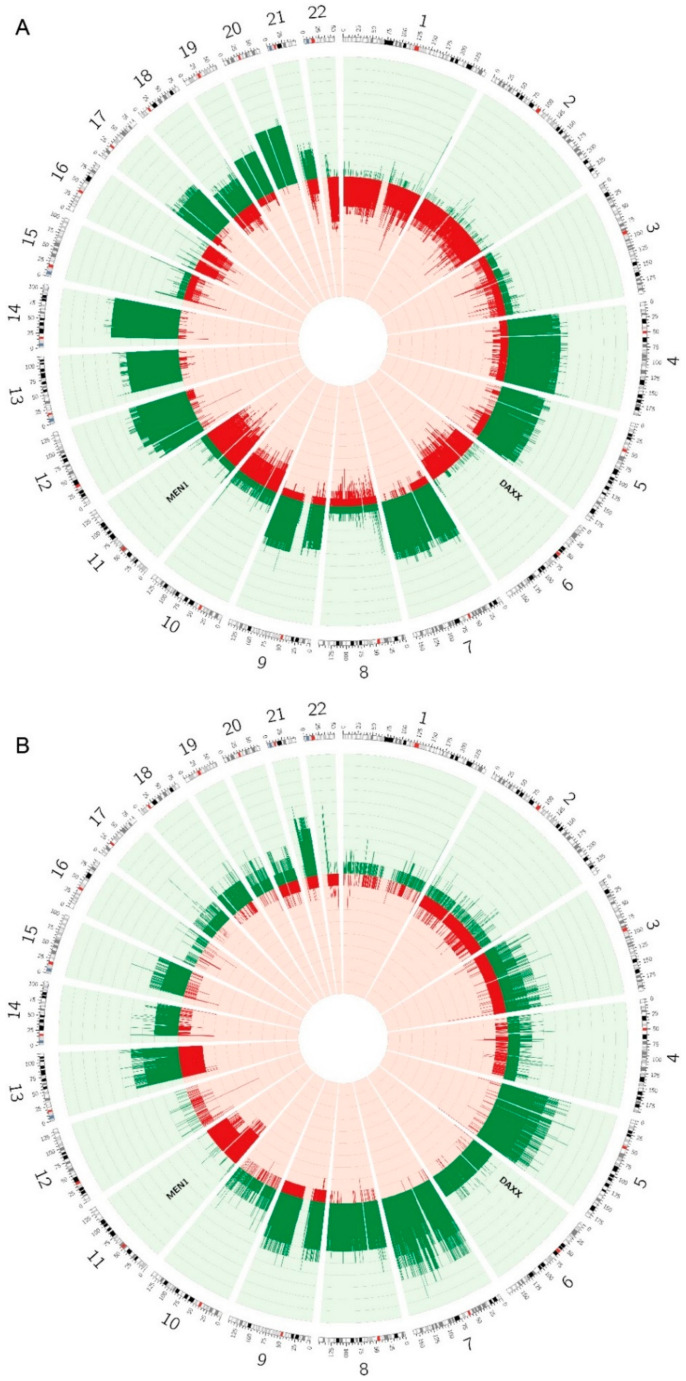
Circos plots of the frequency of copy number alterations observed in PNENs of (**A**) subtype A and (**B**) subtype B. Gains are indicated in green and losses in red.

To further compare the characteristics of the two subtypes, we have compared their copy number alteration (CNA) profiles. Data from methylation arrays can be used to infer CNAs in a similar way as SNP arrays. To perform CNA calling, we used the conumee package which uses normal samples, preferably on the same arrays, as a reference and calculates the log ratio (logR) [26]. We have therefore performed the CNA calling only on the EPIC study cohort, for which good normal control samples and more data points were available, given the large number of probes on the EPIC DNA methylation arrays. To validate CNA calling on the methylation data, we compared the CNA profile based on DNA methylation analysis with the CNA profile based on whole-exome sequencing (WES) analysis for two tumors where both types of data were available [27]. Results were very comparable between CNAs on WES and on the methylation data (Figure S1). Then, the CNA profile was determined for every tumor and the frequencies of gains and losses per subtype were calculated and shown as a circos plot (Figure 5). In the EPIC cohort, 16 tumors were of subtype A and 10 tumors of subtype B.

Overall, the pattern of CNAs seems to differ between tumors of subtype A and tumors of subtype B. Chromosomal losses are more commonly observed in subtype A tumors, with chromosomes 1, 2, 6, 10, 11, 16 and 22 being most affected. The frequency of losses is low in subtype B, however, loss of chr11 is frequently observed and to a lesser extent loss of chr13, which is not observed in subtype A. Chromosomal gains are common in both subtypes and frequencies of gains of several chromosomes, such as chromosomes 5, 7, 9 and 13, seem comparable in both subtypes. In addition, gains are also commonly observed in subtype A in chromosomes 4, 12, 14, 17, 18, 19, 20 and 21. Gains in chr21 seem, however, more frequent in subtype B. Furthermore, in subtype B we have observed gains of chr6, for which we have observed losses in subtype A, and a relatively high frequency of gains in chr8, for which there were barely any chromosomal alterations observed in subtype A.

## 3. Discussion

DNA methylation is an important epigenetic mechanism for the regulation of gene expression and plays a crucial role in cell differentiation. Moreover, changes in DNA methylation have been indicated to play a major role in multiple diseases including cancer, where it promotes tumor development and progression. In PNENs, for example, genes that play a role in epigenetic regulation are frequently mutated, suggesting an important role for epigenetic deregulation in this tumor type [9].

Our study has analyzed genome-wide differential methylation between PNENs and their normal counterparts, the endocrine cells of the pancreas situated in the pancreatic islets. All samples were fresh frozen and analyzed using the EPIC DNA methylation array that interrogates approximately 850,000 CpGs across the genome, which is two times more than most previous studies [10,11,15,28]. Clustering analysis showed that pancreatic islets clustered closely together and separately from PNENs based on their DNA methylation profile. Differential methylation analysis between pancreatic islets and PNENs identified 26,759 DMPs and 79 DMRs. We compared the distribution of hypo- and hypermethylated DMPs over different genomic features. Interestingly, the highest portion of hypomethylated DMPs could be found in intergenic regions (28.8%), which might result from a global hypomethylation that is frequently observed in cancer [29]. The portion of hypermethylated DMPs in IGRs was approximately three times less (10.2%), and this pattern has also been observed by Naumov et al. in colorectal cancer [30]. Furthermore, high portions of hypermethylated DMPs were found in the gene body (22.5%) and the 5’UTR (22.1%) and gene body methylation has been described to be able to increase the gene expression, for example, of metabolic genes or oncogenes [31]. Gene set enrichment analysis has highlighted the MAPK pathway as a possibly important pathway in PNENs, which has been suggested before based on genetic and epigenetic alterations [9,12,32,33]. Cross-talk between the PI3K/AKT/mTOR pathway and the MAPK pathway has been described in neuroendocrine neoplasms and other cancer types, and dual inhibition of these pathways has been shown to be more effective in in vitro and in vivo neuroendocrine neoplasm models [34,35,36]. Despite accumulating evidence implicating the MAPK pathway in PNENs, clinical trials studying the effect of MAPK pathway inhibition are currently lacking [37]. “Telomere maintenance” was another enriched gene set, which is interesting since *DAXX* and *ATRX*, among the most frequently mutated genes in PNENs, play a role in telomere maintenance and since it has been highlighted as one of the four main altered pathways in PNENs [8,9]. In addition, also other epigenetically regulated processes such as chromatin and chromosome condensation are situated in the top GO categories, which again supports the importance of epigenetic deregulation in PNENs. Platelet-related pathways were also enriched, which has been observed previously by Tirosh et al. in familial PNENs [12]. It has been shown that PNEN patients with tumor-associated platelets have a worse prognosis, possibly through modulation of the platelet behavior [38]. Another enriched gene set was olfactory receptors. Although a clear mechanistic link between PNENs and olfactory signaling is currently lacking, there is increasing evidence for a higher expression of olfactory receptors in different cancer tissues [39]. For example, a high expression of OR51E1 has been found in small intestinal neuroendocrine neoplasms [40]. Hence, this could be an avenue worth exploring in further research.

Enrichment analysis for both differential methylation of individual probes and DMRs has highlighted altered DNA methylation in genes related to the immune system. To further explore this, we have evaluated a subset of the EPIC cohort samples for the presence of stromal TILs. In general, a low percentage of TILs (≤5%) was observed with only three samples that had a percentage of TILs between 5% and 30%. This observation is in accordance with the accumulating literature that attempts to unravel the immune landscape of PNENs and which reports a presence of TILs within the tumor microenvironment of PNENs, but mostly in very small numbers [41,42,43]. In addition, the loss of expression of MHC I class molecules and altered expression of immunomodulatory factors has been reported in PNENs and might allow them to evade control of the immune system [41,42]. Several of the master regulator proteins, differentially expressed proteins with an important role in tumorigenesis, that were identified by Alvarez et al. in gastroenteropancreatic NENs (GEP-NENs) were immunomodulatory factors [44]. This also suggests an important role for immune modulation in GEP-NENs and DNA methylation might be a mechanism to regulate the gene expression of these proteins. Multiple studies evaluating immunotherapy in well-differentiated GEP-NENs are ongoing, but so far, observed response rates have been low [45,46]. Possible explanations are the cold immune microenvironment, i.e., the lack of immune cells, which might be caused by the described immune modulation in GEP-NENs resulting in immune evasion, and the low tumor mutational burden in GEP-NENs, as a high tumor mutational burden has been shown to associate with an increased benefit of immunotherapy [47]. Additional integrated studies on epigenomic, transcriptomic and proteomic level towards these immunomodulatory mechanisms in GEP-NENs might identify possible therapeutic targets that allow to prime these tumors for immunotherapy.

Recently, two subtypes of PNENs with different clinical outcomes have been described and these types were suggested to be linked to cell-of-origin [15,16]. Chan et al. distinguished the different types based on mutation status of *ATRX*/*DAXX/MEN1* (A-D-M) and showed that the A-D-M mutated tumors had an alpha cell signature [15]. Cejas et al. used enhancer signatures of chromatin immunoprecipitation sequencing to distinguish two subtypes, which they defined as A and B, and that were alpha or beta cell-like, respectively, based on their expression profiles [16]. In addition, they also distinguished the subtypes using immunohistochemistry for ARX and PDX1, two important transcription factors for alpha and beta cell differentiation, respectively.

As DNA methylation plays a crucial role in cell differentiation and determines the expression of cell type-specific transcription factors, we aimed to assess for the first time whether DNA methylation of the *PDX1* gene region allowed distinguishing the two subtypes and to assess its prognostic value. This analysis was performed in an extensive and global cohort of 83 PNENs, for which long-term follow-up data were present for many. Unsupervised hierarchical clustering based on the *PDX1* methylation data showed two subpopulations, of which, one contained the alpha cells, the A type cluster, and the other contained the beta cells, the B type cluster. An amount of 21 PNENs belonged to subtype B and 62 to subtype A. There was a significant association between mutation status and subtype, with most mutated PNENs being of subtype A, in accordance with the literature [15]. However, two PNENs with *ATRX* mutations, of which, one was subclonal, were of subtype B. Cejas et al. has also observed an alternative lengthening of telomeres (ALT), which is associated with *DAXX* and *ATRX* mutations, in approximately 14% of subtype B PNENs [11,16]. Although *ATRX/DAXX/MEN1* mutations are thus not exclusively observed in PNENs of subtype A, they are clearly more common in this subtype, suggesting that subtype A PNENs are more prone to acquire mutations in these genes. Furthermore, we also identified an association with functional PNENs, linking functional tumors to cell-of-origin, as has been described [14]. The only glucagonoma in our cohort, which is thought to originate from alpha cells, was assigned to subtype A. Eight insulinomas were included in the study, of which, six were assigned to subtype B, which would be expected as they supposedly originate from beta cells based on their hormone production [14]. Of the two subtype A insulinoma patients, one relapsed after a period of 10 years and died of her disease, while the other had a large tumor that had acquired an *ATRX* mutation, which is supposed to be a later stage alteration [11]. Possibly these two subtype A insulinomas were more aggressive. Most insulinomas are indolent tumors, but 10% of the insulinoma patients develop metastasis, which drastically worsens prognosis [48]. Due to the rarity of these tumors, the molecular basis for these differences remains unclear. *YY1* was shown to be recurrently mutated in insulinomas, while mutations in *ATRX/DAXX/MEN1* are very uncommon [49]. Hackeng et al. performed immunohistochemistry for PDX1 and ARX in 37 insulinomas [50]. Five patients were metastatic at diagnosis or developed metastases and one of these was PDX1-negative and they were all ARX-positive, while the others did not express ARX. Transdifferentiation of ARX-positive PNENs to insulin-producing PNENs could provide an explanation for these observations and the more malignant behavior of these exceptional insulinomas [50,51,52]. The identification of PNENs that produce different combinations of neuroendocrine hormones and the observation that the RNA expression of hormones can differ between primary tumor and metastases supports this hypothesis that assumes that specific hormone production can become disrupted [15,21]. The other functional PNENs in our study are divided over the two subtypes. For an improved understanding of their cell-of-origin, it would be interesting to determine the genome-wide DNA methylation profile of all endocrine cell types in the pancreas of which data are currently lacking.

The presence of distant metastasis at diagnosis and recurrence after surgery are significantly more common for subtype A PNENs, and a trend towards significance was observed for LVI at diagnosis. The subtypes did not differ regarding WHO grade or tumor size. Furthermore, CoxPH modeling showed that subtype B had a better overall survival compared with subtype A (HR: 0.22 [95% CI: 0.051–0.95], *p* = 0.043). This is in line with previous studies that report a better outcome in A-D-M WT PNENs and B type PNENs [15,16]. In addition, CoxPH analysis in a patient subset without distant metastasis at diagnosis showed a trend towards significance (HR of 0.16 [95% CI: 0.019–1.4], *p* = 0.092), hinting that the observed effect of subtype on survival is not only due to its association with the presence of metastasis at diagnosis.

CNA profiles were generated for the two subtypes, based on EPIC array data of 26 PNENs. The observed chromosomal alterations often entailed whole chromosomes and the affected chromosomes matched with previous reports [53,54]. The comparison of the CNA profiles of both subtypes suggested a difference in CNA pattern. Interestingly, subtype A PNENs had losses in chr6, which contains the *DAXX* gene which is frequently mutated in subtype A tumors, while gains of this chromosome were observed in subtype B PNENs. In the COSMIC cancer census gene list (COSMIC v91), no amplifications in genes on chr6 have been linked to cancer. However, deletion of *PRDM1* and *TNFAIP3* on chr6 has been linked to cancer [55]. In addition, subtype B PNENs frequently had gains in chr8, while alterations in this chromosome were rarely observed in subtype A PNENs. In the COSMIC cancer census gene list, the *MYC, NSD3* and *KAT6A* genes on chr8 have been amplified in cancer [55]. Of these three genes, only *MYC* has been studied in PNENs [56]. MYC is an oncogenic transcription factor which was found highly expressed in 81% of PNEN patients. It is a downstream target of the AKT/mTOR pathway, but also seemed to upregulate this pathway through negative regulation of PTEN. Therefore, it was suggested that the MYC expression might be a resistance mechanism against everolimus treatment and that MYC might be a potential therapeutic target [56]. The differences in CNA pattern and A-D-M mutations between the two subtypes might suggest alternative molecular driver mechanisms. However, molecular analysis in a larger cohort and with a dedicated technique, such as whole-genome sequencing, might provide an improved and more detailed understanding of the molecular profile of the subtypes. In addition, Lawrence et al. showed that copy number profiling can also be used to perform subtyping in PNENs, therefore, it might be interesting to combine both approaches in a follow-up study [21].

Our study has validated DNA methylation profiling of *PDX1* as an alternative method to perform subtyping of PNENs. The two identified subtypes have a different prognosis and a different risk of relapse, which would support a different strategy in clinical follow-up, with a closer follow-up for subtype A patients. In addition, also at the molecular level, the two subtypes seem different, with different CNA patterns and with *ATRX/DAXX/MEN1* mutations more commonly observed in subtype A PNENs. Possibly, the molecular driver mechanisms in these PNEN subtypes differ, which could require different treatment strategies, but further molecular and clinical research is required.

## 4. Materials and Methods 

### 4.1. Study Population

Patients that were diagnosed between 2003 and 2018 with a well-differentiated PNEN and that underwent surgery for their tumor were included. All patients were older than 18 years of age. Residual tumor tissue was snap-frozen after surgery and stored at −80 °C. The Belgian Virtual Tumorbank was used to identify Belgian centers that had these samples available [20]. Samples were collected in Belgium from the biobank at the Antwerp University Hospital (biobank@UZA, Antwerp, Belgium; ID: BE71030031000; Belgian Virtual Tumourbank funded by the National Cancer Plan, BBMR-ERIC; No. Access: 3, Last: August, 22, 2018) [19], the "biothèque" of the University of Liège (BUL)—University Hospital (CHU) Liège and the tumor bank of the University Hospital Brussels (UZ Brussel) and in New Zealand from the Cancer Society Tissue Bank, University of Otago, NZ and Auckland Region Hospitals [21]. In addition, clinicopathological data of the included patients were collected. WHO grade is reported according to the WHO 2017 classification system [57].

Fresh frozen pancreatic islets from 5 different donors were purchased from Prodo Laboratories Inc., which provides pancreatic islets with research consent from Organ Procurement Organizations (OPOs). All human islet samples had a viability of 95% and a purity of 85%–95%.

The study was approved by the local ethics committee of Antwerp University Hospital/University of Antwerp (approval number 16/46/490) and by the New Zealand Health and Disability Ethics committee (approval numbers 13/NTA/69 and 13/NTB/173).

### 4.2. Tissue Procurement and Nucleotide Extraction

For all tumor blocks, a fresh frozen hematoxylin-eosin (HE)-stained section was reviewed by a dedicated pathologist to confirm diagnosis and estimate the tumor cell percentage and for a subset to evaluate the presence of stromal tumor-infiltrating lymphocytes (TILs) (according to the International TILs Working Group recommendations [58]). Only samples with a tumor cell percentage higher than 60% were included. Fifteen 10 µm slides of fresh frozen tumor tissue or 1000 islet equivalents (IEQs) were used as the input for the DNA isolation using the AllPrep DNA/RNA Micro kit (Qiagen, Hilden, Germany). DNA quality and DNA concentration were measured using the NanoDrop (Thermo Scientific, Wilmington, DE, USA) and Qubit 2.0 fluorometer with the dsDNA Broad-Range Assay (Thermo Scientific), respectively.

Sample processing of the New Zealand cohort has been described previously [21].

### 4.3. Mutation Analysis

Whole-exome sequencing of 22 patients (UZA-01–UZA-10 and Lawrence-001–Lawrence-012) and deep targeted sequencing of 3 patients (Lawrence-013–Lawrence-015) has been performed in previous studies [21,27]. The identified variants in *MEN1*, *DAXX* and *ATRX* were validated using Sanger sequencing, if the allelic ratio was high enough, and are summarized in Table 2.

### 4.4. DNA Methylation Analysis

An amount of 500 ng of DNA was used as the input for bisulfite conversion using the EZ DNA methylation kit (Zymo Research, Freiburg, Germany). The 26 Belgian samples and the 15 New Zealand were subjected to genome-wide DNA methylation analysis using the Infinium MethylationEPIC array (Illumina, San Diego, CA, USA) and the Infinium HumanMethylation450K BeadChips (Illumina), respectively. The iScan system (Illumina) was used to scan the arrays, generating raw IDAT files.

### 4.5. Additional DNA Methylation Data

Infinium HumanMethylation450K BeadChips (Illumina) DNA methylation data of additional PNEN patients and alpha and beta cell samples were collected from public repositories (Figure 1). Neimann et al. isolated alpha and beta cell samples from, respectively, two and three donors and subjected the samples to DNA methylation analysis [17]. Furthermore, DNA methylation data were obtained from 32 PNENs analyzed by Chan et al. [15], 5 PNENs analyzed by Timp et al. [22] and 5 PNENs analyzed within the PAAD project of the TCGA Research Network (https://www.cancer.gov/tcga). We obtained available clinicopathological data and included the mutation status of the *ATRX*, *DAXX* and *MEN1* genes, if available.

### 4.6. Exploratory DNA Methylation Data Analysis

Data analysis was performed in R version 3.5 for the analysis with ChAMP and version 3.6 for the other analyses. Read-in of the raw IDAT files was performed using the Minfi package [59], and for further analysis, including quality control, filtering and normalization, the ChAMP package version 2.12.2 was implemented [23]. After data read-in, methylation values for CpGs were calculated, which are represented as beta values between 0 and 1, with a value of 0 being not methylated and a value of 1 being completely methylated. Next, filtering was performed using ChAMP, including filtering of underperforming probes, which can be due to the presence of single nucleotide polymorphisms (SNPs), non-unique mapping or off-target hybridization, based on Zhou et al. [60]. Normalization using the BMIQ method was performed to correct for the differences in dynamic range between the two probe technologies used for the DNA methylation arrays [61]. The normalized beta values were used for further analysis. Singular value decomposition (SVD) analysis within the ChAMP package was used to determine which parameters contribute to variability in the data, as the cut-off of significance to identify technical sources of variation 0.01 was used. ChAMP implements the limma package, which uses linear modelling to calculate a *p*-value for differential methylation of individual probes, to identify differentially methylated probes (DMPs) [62]. Benjamini–Hochberg multiple testing correction was applied to the *p*-values and an adjusted *p*-value ≤ 0.05 was required for a CpG to be identified as a DMP. To identify differentially methylated regions (DMRs), extended genomic regions with a quantitative difference in DNA methylation between two groups, the default method in ChAMP, namely Bumphunter, was used [63]. Settings were default, except the minimal number of probes which was set at 3. Gene set enrichment analysis was carried out using the methylRRA function with method GSEA of the methylGSA package, which adjusts for the number of CpGs in each gene [24]. Analysis was done for the Reactome, Gene Ontology (GO) and KEGG gene sets. For annotation and gene set enrichment analysis of DMRs, the Genomic Regions Enrichment of Annotations Tool (GREAT) v3.0.0 was used [25].

### 4.7. PDX1 Data Analysis

All CpGs situated in the region of the *PDX1* gene (chr13:28480000–28510000), as described by Cejas et al. [16], that are common between 450K and EPIC arrays, were selected. Normalized beta values of all tumor samples and alpha and beta cells for these CpGs were used to perform unsupervised hierarchical clustering analysis with pvclust [64]. Euclidean distance was used as the distance measure, Ward’s minimum variance as the method for hierarchical clustering and 1000 bootstrap replications were performed. The dendextend package was used for generating the dendrogram [65].

### 4.8. Copy Number Alteration Calling

Identification of copy number alterations (CNAs) was performed using the conumee package v1.18 in R with default settings, which has a minimal binsize of 50 kb [26]. Then, circos plots were created to visualize the frequency of gains (log2 ratio ≥ 0.150) and losses (log2 ratio ≤ −0.150) of groups of samples [66].

### 4.9. Statistical Analysis

Statistical analysis was performed in R version 3.6. Fisher’s exact test was used to test associations between two categorical variables. The *t*-test was used to determine differences in normally distributed continuous variables between two groups and the Mann–Whitney test for non-normally distributed continuous variables. Overall survival analysis was performed via univariate and multivariate Cox proportional hazards regression modeling and proportionality was confirmed using Schoenfeld residuals. The obtained *p*-values were plotted on Kaplan–Meier survival curves. All *p*-values were based on two-sided hypothesis testing and 0.05 was used as the cut-off for statistical significance.

## 5. Conclusions

PNENs have a genome-wide DNA methylation profile that is different from normal pancreatic islets. Enrichment analysis has indicated several pathways that are affected by these DNA methylation alterations, including the MAPK pathway and immune system-related pathways. In addition, we showed for the first time that, besides PDX1 expression, also the DNA methylation profile of the *PDX1* gene region can be used to distinguish two PNEN subtypes, A and B, and illustrate a link to their respective cell-of-origin, alpha and beta cells. The two subtypes seem to have different clinicopathological characteristics, a different molecular profile and a different prognosis, with significantly worse outcomes in subtype A PNENs.

## Figures and Tables

**Table 1:** Association between clinicopathological characteristics and subtype.

Clinicopathological Parameter	Subtype A	Subtype B	*p*-Value
Gender			0.20
F	25	13	
M	32	8	
Mutation *MEN1/ATRX/DAXX*			7.6E-06
No	10	13	
Yes	38	2	
WHO Grade			0.66
G1	27	12	
G2	22	8	
G3	3	0	
Functionality			0.011
Gastrinoma	1	1	
Glucagonoma	1	0	
Insulinoma	2	6	
Non-functional	46	13	
VIPoma	2	0	
LVI			0.059
No	14	8	
Yes	31	5	
Distant metastasis			0.022
No	32	18	
Yes	21	2	
Recurrence			5.9E-04
No	14	16	
Yes	13	0	
Age	57.1 (±12.1)	53.1 (±15.0)	0.29
Tumor size	4.4 (±2.6)	3.77 (±3.2)	0.18

The counts are shown for categorical variables and the mean (±standard deviation) for continuous variables. Significant *p*-values (≤ 0.05) are indicated in bold. (LVI, lymphovascular invasion).

**Table 2:** Overview of mutations in *DAXX, ATRX* or *MEN1* identified in 25 patients (UZA-01–UZA-10 and Lawrence-001–Lawrence-015).

Patient	Gene	Mutation	Allelic Ratio WES	Sanger Validated?
UZA-01	*MEN1*	chr11:64572212 CG > C (frameshift)	100%	Yes
UZA-01	*MEN1*	chr11:64571924 G > T (stopgain)	11%	No
UZA-03	*DAXX*	chr6:33288573 G > A (stopgain)	60%	Yes
UZA-06	*DAXX*	chr6:33289250 G > A (missense)	8%	No
UZA-07	*MEN1*	chr11:64575561 C > A (missense)	53%	Yes
UZA-07	*DAXX*	chr6:33287917 C > A (stopgain)	48%	Yes
UZA-08	*ATRX*	chrX:76938655 T > C (missense)	5%	No
Lawrence-001	*MEN1*	chr11:64577293 T/TC (frameshift)	/	Yes
Lawrence-002	*MEN1*	chr11:64575561 C/CA (frameshift)	/	Yes
Lawrence-003	*MEN1*	chr11:64575530 C/T (missense)	/	Yes
Lawrence-004	*MEN1*	chr11:64573723 TGTCCGCCCAGGC/T (in-frame deletion)	/	Yes
Lawrence-004	*ATRX*	chrX:76944420 TCTAGGAGAAAGGA/T (splicing)	/	Yes
Lawrence-006	*MEN1*	chr11:64572613 G/GCAA (in-frame insertion)	/	Yes
Lawrence-008	*MEN1*	chr11:64573744 CT/C (frameshift)	/	Yes
Lawrence-009	*ATRX*	chrX:76813035 C/A (stopgain)	/	Yes
Lawrence-011	*MEN1*	chr11:64572078 63Nucleotides/G (frameshift)	/	No
Lawrence-011	*DAXX*	chr6:33287491 33Nucleotides/C (frameshift)	/	Yes
Lawrence-012	*MEN1*	chr11:64577329 TAGAC/T (frameshift)	/	Yes
Lawrence-012	*ATRX*	chrX:76778831 CTACAAT/C (in-frame deletion)	/	Yes
Lawrence-013	*MEN1*	chr11:64572643 G/A (stopgain)	/	Yes
Lawrence-014	*MEN1*	chr11:64575550 C/A (missense)	/	Yes

## Supplementary Materials

The following are available online at https://www.mdpi.com/2072-6694/12/6/1461/s1, Figure S1: Comparison of copy number alteration profiles of (**A**) UZA-03 and (**B**) UZA-07 as determined via whole-exome sequencing (WES, top) and DNA methylation analysis (Methylation, bottom), Table S1: Clinicopathological data of all included patients, Table S2: Top 10 differentially methylated probes, Table S3: Results of the gene set enrichment analysis (Reactome, Gene Ontology (GO) and KEGG).
