# Supplementary material for: PDX1 DNA Methylation Distinguishes Two Subtypes of Pancreatic Neuroendocrine Neoplasms with a Different Prognosis
Source: Cancers (Basel). 2020 Jun 4;12(6):1461. doi: 10.3390/cancers12061461 (PMC7352978; doi:10.3390/cancers12061461)
Supplement: Supplementary file 1 [file cancers-12-01461-s001.zip › cancers-816999 supplementary/cancers-816999 supplementary figure.pdf]

cancers

MDPI

PDX1 DNA Methylation Distinguishes Two Subtypes of Pancreatic Neuroendocrine Neoplasms with a Different Prognosis

Gitta Boons [1,2], Timon Vandamme [1,2,3,4], Joe Ibrahim [1,2], Geert Roeyen [5], Ann Driessen [6], Dieter Peeters [7,8], Ben Lawrence [9,10], Cristin Print [10,11], Marc Peeters [1], Guy Van Camp [1,2] and Ken Op de Beeck [1,2,*]

[1] Center for Oncological Research, University of Antwerp and Antwerp University Hospital, Antwerp 2610, Belgium
[2] Center of Medical Genetics, University of Antwerp and Antwerp University Hospital, Edegem 2650, Belgium
[3] Section of Endocrinology, Department of Internal Medicine, Erasmus Medical Center, Rotterdam 3015GD, Netherlands
[4] NETwerk, Antwerp University Hospital, Edegem 2650, Belgium
[5] Department of Hepatobiliary, Endocrine and Transplantation Surgery, Antwerp University Hospital, Edegem 2650, Belgium
[6] Department of Pathology, Antwerp University Hospital and University of Antwerp, Edegem 2650, Belgium
[7] Histopathology, imaging and quantification unit, HistoGeneX, Antwerp 2610, Belgium
[8] Department of Pathology, AZ Sint-Maarten, Mechelen 2800, Belgium
[9] Discipline of Oncology, Faculty of Medicine and Health Sciences, University of Auckland, Auckland 1023, New Zealand
[10] Maurice Wilkins Centre hosted by the University of Auckland, Auckland 1023, New Zealand
[11] Department of Molecular Medicine and Pathology, School of Medical Sciences, Faculty of Medicine and Health Sciences, University of Auckland, Auckland 1023, New Zealand
* Correspondence: ken.opdebeeck@uantwerpen.be; Tel.: +32-3275-97-91

Supplementary

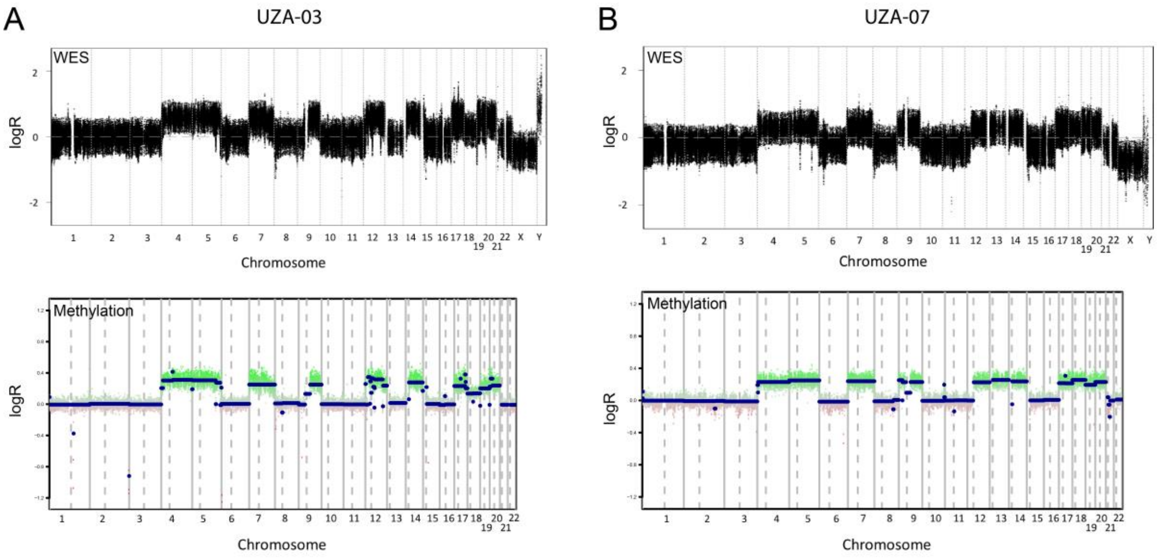

Figure S1. Comparison of copy number alteration profiles of (**A**) UZA-03 and (**B**) UZA-07 as determined via whole-exome sequencing (WES, top) and DNA methylation analysis (Methylation, bottom).
